# Quasi-probability information in an coupled two-qubit system interacting non-linearly with a coherent cavity under intrinsic decoherence

**DOI:** 10.1038/s41598-020-70209-5

**Published:** 2020-08-06

**Authors:** Abdel-Baset A. Mohamed, Hichem Eleuch

**Affiliations:** 1 Department of Mathematics, College of Science and Humanities in Al-Aflaj, Prince Sattam bin Abdulaziz University, Al-Aflaj, Saudi Arabia; 2 Department of Mathematics, Faculty of Science, Assiut University, Assiut, Egypt; 3 Department of Applied Sciences and Mathematics, College of Arts and Sciences, Abu Dhabi University, Abu Dhabi, UAE; 4 Institute for Quantum Science and Engineering, Texas A&M University, College Station, TX 77843 USA

**Keywords:** Quantum information, Qubits, Single photons and quantum effects

## Abstract

We explore the phase space quantum effects, quantum coherence and non-classicality, for two coupled identical qubits with intrinsic decoherence. The two qubits are in a nonlinear interaction with a quantum field via an intensity-dependent coupling. We investigate the non-classicality via the Wigner functions. We also study the phase space information and the quantum coherence via the Q-function, Wehrl density, and Wehrl entropy. It is found that the robustness of the non-classicality for the superposition of coherent states, is highly sensitive to the coupling constants. The phase space quantum information and the matter-light quantum coherence can be controlled by the two-qubit coupling, initial cavity-field and the intrinsic decoherence.

## Introduction

Quantum effects, quantum coherence and non-classicality, of two-level systems (qubits) are key features of quantum physics. They are operational resources, in modern applications in quantum technology^1^, as: quantum algorithms^2^, quantum computation^3^, and quantum key distribution^4^. Moreover, the quantum effects and quantum correlations have been explored, both theoretically^5–9^ and experimentally^10^. Recently, due to the rapid development of the real qubit systems based on the superconducting circuits^11^ and quantum dots^12^, the quantum effects have been further investigated^13–15^.

Phase-space distributions, Wigner function (WF)^16^ and Q-function (QF)^17^, are important tools to investigate the quantum effects. The WF distribution^18,19^ is a powerful appliance to explore the non-classicality via the positivity and negativity of the Wigner function. The negativity of WF is a sufficient but not necessary condition for non-classicality^20–23^. Phase space non-classicality can be visualized through the negative part of WF distribution, which cannot occur for classical light. It is a necessary and sufficient identifier for the experimental reconstruction of an entanglement quasiprobability^10,24^.

The QF distributions are always positive distributions^25^, and they are useful for exploring the phase-space information, coherence and entanglement. Entanglement and coherence^26^ are crucial resources for quantum information^27^. Based on the QF, Wehrl density and Wehrl entropy^28^ are introduced to quantify the phase space information and the entanglement^29^. These QF quantifiers were studied only for the phase space of the cavity fields, one-qubit^25,29^ and one-qutrit^30,31^. Whereas, in the multi-qubit phase space, the QF, and its applications are still in need of more investigation.

The quantum information entropies [von Neumann entropy^32^, linear entropy^33^] are used to measure the quantum coherence. Wehrl entropy delivers a valuable phase space information on the purity and the entanglement. The dynamics of the Wehrl entropy and the von Neumann entropy are very similar. Without decoherence, the von Neumann entropy, linear and Wehrl entropies of a bipartite-system are used to measure the entanglement between the two sub-systems^12^. Whereas, with the decoherence, the Wehrl entropy is used only to measure the phase space purity-loss of one of them^34^.

The quantum effects, quantum coherence and non-classicality, deteriorate due to the decoherence effects. The intrinsic decoherence (ID)^35^ is one of the phenomena which responsible of the coherence destruction. This ID model is previously applied to the two-qubit system in linear interaction with a cavity field^36^, where the intensity-dependent coupling and the coupling between the qubits are neglected.

Motivated by the important role of the phase space quantum effects, intrinsic decoherence and coherent fields in the quantum information, we introduce analytical solutions for the intrinsic decoherence model of two coupled qubits nonlinearly interacting with a coherent cavity-field. Therefore, the dynamics of the non-classicality, the phase space information and the quantum coherence will be analyzed based on the quasi-probability distributions.

In “Physical model and density matrix” section, the physical intrinsic decoherence model and the dynamics of the density matrix are presented. While the quasi-probability WF distribution is considered in “Wigner distribution” section. In “Q-distribution” section we examine the Q-distribution and its associated measures. We conclude our investigation in “Conclusion” section.

## Physical model and density matrix

### Hamiltonian

Here, we considered two coupled identical qubits that are interacting nonlinearly with a quantum cavity-field (with the same frequency $\omega$) via intensity-dependent coupling. This system can be realized as an artificial atomic system (such as superconducting qubits with a resonator^37^ or with LC circuit^38^), in addition to the atomic systems (such as atoms interacting with a cavity field^39^, nuclear spins interacting with a magnetic field^40,41^).

In the resonant case and using the rotating wave approximation, the Hamiltonian of the total system, in units of $\hbar$, is

1 $$\begin{aligned} \hat{H}= & {} \omega \hat{a}^{\dag }\hat{a}+\omega (\hat{\sigma }_{z}^{(1)}+\hat{\sigma }_{z}^{(2)}) +\lambda [ \hat{a}\hat{A}(\hat{\sigma }^{(1)}_{+}+\hat{\sigma }^{(2)}_{+}) \nonumber \\&+ \hat{A}^{\dag }\hat{a}^{\dag }(\hat{\sigma }^{(1)}_{-} +\hat{\sigma }^{(2)}_{-})]+J(\sigma ^{(1)}_{+}\sigma ^{(2)}_{-} +\sigma ^{(2)}_{+}\sigma ^{(1)}_{-}), \end{aligned}$$

$\hat{\sigma }^{(i)}_{\pm }$ and $\hat{\sigma }^{(i)}_{z}$ represent the Pauli matrices which can be expressed in the bases formed by the excited states $|e_{i}\rangle$, and ground states $|g_{i}\rangle$ as: $\hat{\sigma }^{(i)}_{+}=|e_{i}\rangle \langle g_{i}|$, $\hat{\sigma }^{(i)}_{-}=|g_{i}\rangle \langle e_{i}|$ and $\hat{\sigma }^{(i)}_{z}=|e_{i}\rangle \langle e_{i}|-|g_{i}\rangle \langle g_{i}|$. The qubits and the cavity-field have the same frequency $\omega$. *J* designs the interaction coupling constant between the two qubits. The operator $\hat{A}=\sqrt{\hat{a}^{\dag }\hat{a}}$ represents the intensity-dependent operator.

In the space states $\{ \,|1\rangle _{n}=|e_{1}e_{2},n\rangle , |2\rangle _{n}=|e_{1}g_{2},n+1\rangle , |3\rangle _{n}=|g_{1}e_{2},n+1\rangle , |4\rangle _{n}=|g_{1}g_{2},n+2\rangle \,\}$, the dressed states $|\Psi ^{n}_{i}\rangle$ and their eigenvalues of Eq. (1) are given by

2 $$\begin{aligned} \!\!\!\!\left( \begin{array}{c} |\Psi ^{n}_{1}\rangle \\ |\Psi ^{n}_{2}\rangle \\ |\Psi ^{n}_{3}\rangle \\ |\Psi ^{n}_{4}\rangle \\ \end{array} \right)= & {} \text {[M]} \left( \begin{array}{c} |1\rangle _{n} \\ |2\rangle _{n} \\ |3\rangle _{n} \\ |4\rangle _{n} \\ \end{array} \right) , \nonumber \\&\text {[M]}=\!\!\!\left( \begin{array}{cccc} y_{n} &{} 0 &{} 0 &{} -x_{n}\\ 0 &{} \frac{1}{\sqrt{2}} &{} -\frac{1}{\sqrt{2}} &{} 0 \\ \alpha _{n}^{-}&{} \chi _{n}^{-}&{} \chi _{n}^{-}&{} \beta _{n}^{-} \\ \alpha _{n}^{+}&{} \chi _{n}^{+}&{} \chi _{n}^{+}&{} \beta _{n}^{+} \\ \end{array} \right) , \end{aligned}$$

and

3 $$\begin{aligned} E^{n}_{1}= & {} \omega (n+1), \qquad E^{n}_{2}=\omega (n+1)-J,\nonumber \\ E^{n}_{3}= & {} \omega (n+1)+\frac{1}{2}(J-Z_{n}),\nonumber \\ E^{n}_{4}= & {} \omega (n+1) +\frac{1}{2}(J+Z_{n}), \end{aligned}$$

with,

$$\begin{aligned} x_{n}= & {} \frac{n+1}{W_{n}},\quad y_{n}=\frac{n+2}{W_{n}}, \quad \alpha _{n}^{\pm }=\frac{2\lambda (n+1)}{\sqrt{Z_{n}(Z_{n}\pm J)}},\\ \beta _{n}^{\pm }= & {} \frac{2\lambda (n+2)}{\sqrt{Z_{n}(Z_{n}\pm J)}},\quad \chi _{n}^{\pm }=\pm \sqrt{\frac{Z_{n}\pm J}{4Z_{n}}},\\ Z_{n}= & {} \sqrt{J^{2}+8\lambda ^{2}W_{n}^{2}},\quad W_{n}=\sqrt{(n+1)^{2}+(n+2)^{2}}. \end{aligned}$$

### Intrinsic decoherence model

The dynamics of the master equation is described by^35^

4 $$\begin{aligned} \frac{d \hat{\rho }(t)}{d t}= & {} -i[\hat{H},\hat{\rho }(t)]-\gamma [\hat{H},[\hat{H},\hat{\rho }(t)]], \end{aligned}$$

$\gamma$ represents the ID rate. For the sake of simplicity, we take here $\hbar =1$.

By using Eq. (4), the dynamics of the dressed state matrices, $\Lambda ^{mn}_{ij}(0)=|\Psi ^{m}_{i}\rangle \langle \Psi ^{n}_{j}|_{t=0}$, is given by

5 $$\begin{aligned} \Lambda ^{mn}_{ij}(t)=e^{-i\lambda (E^{m}_{i}-E^{n}_{j})t}D_{F}\Lambda ^{mn}_{ij}(0), \end{aligned}$$

where, $D_{F}=e^{-\gamma (E^{m}_{i}-E^{n}_{j})^{2}t}$ is the ID term.

We focus on the case where the initial state of the two qubits is $\hat{\rho }^{Q_{s}}(0)=|e_{1}e_{2}\rangle \langle e_{1}e_{2}|$, and the cavity is considered initially in a superposition of coherent states,

6 $$\begin{aligned} |\psi (0)\rangle _{C}=\frac{1}{\sqrt{A}}[|\alpha \rangle +r|-\alpha \rangle ]=\sum _{n=0}^{\infty }\eta _{n} \,|n\rangle , \end{aligned}$$

$|\alpha \rangle$ is the coherent state with the mean photon number $|\alpha |^{2}$. The photon distribution function $\eta _{n}$ is given by

$$\begin{aligned} \eta _{n}= \frac{[1+r(-1)^{n}]\alpha ^{n}e^{-\frac{1}{2}|\alpha |^2}}{(1+r^{2}+2\langle \alpha |-\alpha \rangle )\sqrt{n!}}. \end{aligned}$$

where $A=1+r^{2}+2r e^{-2|\alpha |^{2}}$. The parameter *r* takes the values $-1$, 0 and 1 to get the odd coherent, coherent and even coherent states, respectively. The advantage of using the coherent states results in the fact that they are easy to be implemented and widely used in realistic physical systems^42–46^. In the dressed state representation based on the basis $|\Psi ^{n}_{i}\rangle$ we have

7 $$\begin{aligned} \hat{\rho }(0)= & {} \sum ^{\infty }_{m,n=0}\eta _{m}\eta _{n}^{*}\,\, [M^{m}_{11}|\Psi ^{m}_{1}\rangle +M^{m} _{31} |\Psi ^{m}_{3}\rangle +M^{m} _{41} |\Psi ^{m}_{4}\rangle ] \nonumber \\&\qquad \quad \otimes [M^{n} _{11}\langle \Psi ^{n}_{1}|+M^{n} _{31} \langle \Psi ^{n}_{3}|+M^{n} _{41} \langle \Psi ^{n}_{4}|] \end{aligned}$$

From Eqs. (5) and (7), we obtain the following density matrix expression

8 $$\begin{aligned} \hat{\rho } (t)= & {} \sum ^{\infty }_{m,n=0}\eta _{m}\eta _{n}^{*}\,\,\bigg \{ M_{11} M_{11}\Lambda ^{mn}_{11}(t) +M_{31} M_{11}\Lambda ^{mn}_{31}(t) \nonumber \\&\quad +M_{41} M_{11}\Lambda _{41}(t)+M_{11} M_{31}\Lambda _{13}(t) +M_{31} M_{31}\Lambda _{33}(t) \nonumber \\&\quad +M_{41} M_{31} \Lambda _{43}(t) +M_{11} M_{41}\Lambda _{14}(t)+M_{31} M_{41}\Lambda _{34}(t) \nonumber \\&\quad +M_{41} M_{41}\Lambda _{44}(t)\,\,\bigg \}, \end{aligned}$$

where $M_{ij}$ are the elements of the matrix $[\text {M}]$ of Eq. (2). The non-classicality and quantum coherence of the different system partitions, the cavity-field system $\rho ^{C}(t)$ and the two qubits $\hat{\rho }^{Q_{s}}(t)$, will be studied via the Wigner- and Q-distributions. The cavity-field and the two-qubit system are respectively represented, in the cavity-field system basis states $\{|n\rangle \}$ and the two-qubit basis states $\{|\varpi _{1}\rangle =|e_{1}e_{2}\rangle , |\varpi _{2}\rangle =|e_{1}g_{2}\rangle , |\varpi _{3}\rangle =|g_{1}e_{2}\rangle , |\varpi _{4}\rangle =|g_{1}g_{2}\rangle \}$, as:

9 $$\begin{aligned} \!\!\!\!\rho ^{C}(t)= & {} \sum _{j=1}^{4}\langle \varpi _{j}|\hat{\rho }(t)|\varpi _{j}\rangle =\sum ^{\infty }_{m,n=0}\rho _{mn}^{f}|m\rangle \langle n|, \end{aligned}$$

10 $$\begin{aligned} \hat{\rho }^{Q_{s}}(t)=\text {Tr}_{\text {cavity}}\{\rho (t)\} =\sum _{n=0}^{\infty } \langle n|\hat{\rho }(t)|n\rangle . \end{aligned}$$

The reduced density matrices of the *k*-qubit ($k= 1, 2$) are defined by $\rho ^{Q_{1}(Q_{2})}(t)= \text {Tr}_{Q_{2}(Q_{1})}\{\rho ^{Q_{s}}(t)\}$.

## Wigner distribution

The phase space quasi-probability distributions (QPDs) are the measure of the non-classicality for the state $\hat{\rho }(t)$, which are defined by^47,48^:

11 $$\begin{aligned} F(\beta ,s)=\frac{2}{\pi }\sum _{n=0}^{\infty }(-1)^{n} \frac{(1+s)^{n}}{(1-s)^{n+1}}\langle \beta , n|\hat{\rho }(t)|\beta ,n\rangle , \end{aligned}$$

If the parameter $s=0, -1$, we get the Wigner and the Q-distributions, respectively. $|\alpha ,n\rangle =e^{(\alpha \hat{a}^{+}-\alpha ^{*}\hat{a})}|n\rangle$ represents the displaced state number. It is known that the phase space QPDs are built on the density matrix elements. Therefore, the phase space information can be given by the QPDs.

**Figure 1:**
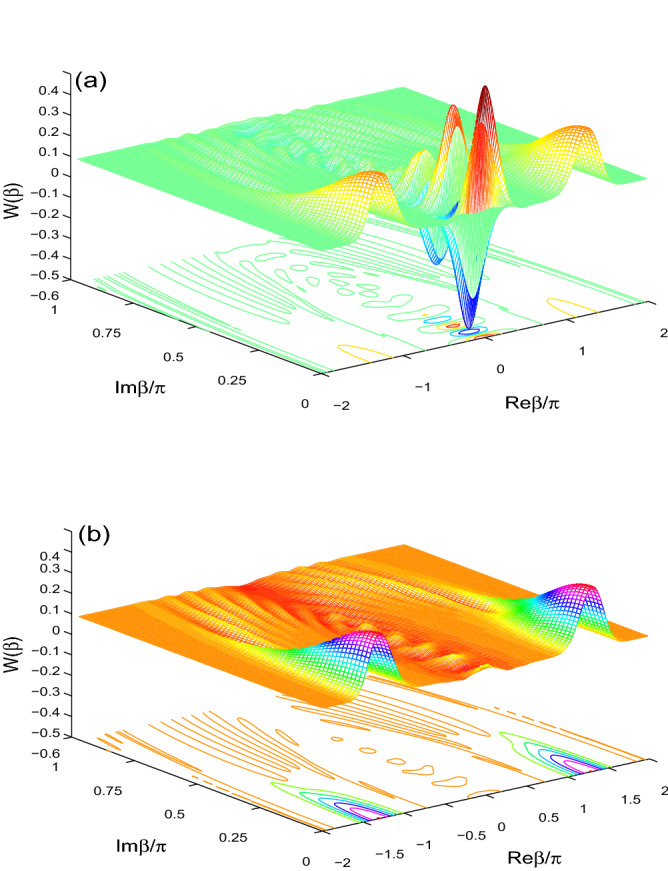
The Wigner function $W(\beta )$ is plotted with $\alpha =4$ at $\lambda t=\pi$ in (**a**) and at $\lambda t=\frac{3}{2}\pi$ in (**b**) for $(J, \gamma )=(0, 0)$.

In the representation of the field coherent state $|\beta \rangle$, the WF of the cavity field is given by^47–49^

12 $$\begin{aligned} W(\beta )&= \frac{2}{\pi }\sum _{p=0}^{\infty } (-1)^{p}\sum _{m,n=0}^{\infty }\frac{p!|\beta |^{-2p}e^{-|\beta |^{2}}}{\sqrt{m! n!}}\beta ^{*m}\beta ^{n} \nonumber \\&\quad \times \, L_{m}^{m-p}(|\beta |^{2})\rho _{mn}^{C}L_{n}^{n-p}(|\beta |^{2}), \end{aligned}$$

$L_{n}^{m-n}(|\beta |^{2})$ represents the associated Laguerre polynomial. The positivity of the WF of a quantum state is an indicator to its minimization uncertainty, while, its negativity indicates the existence of the quantum correlation^50^ due to interference terms in $W(\beta )$. It is also used to explore the classical-quantum boundary.

**Figure 2:**
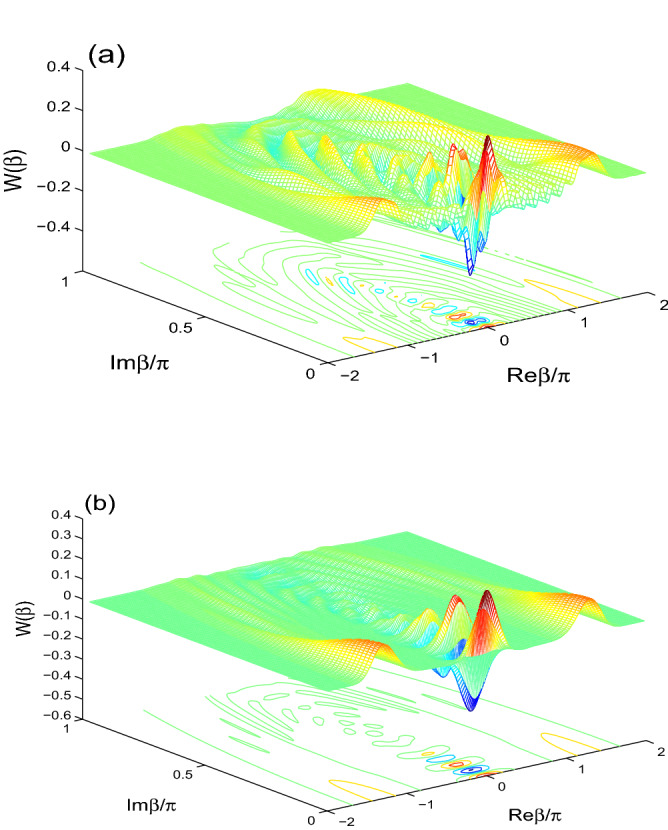
$W(\beta )$ is displayed for $\alpha =4$ at $\lambda t=\frac{3}{2}\pi$ for $(J,\gamma )=(30\lambda , 0)$ in (**a**) and $(J,\gamma )=(0,0.1\lambda )$ in (**b**).

**Figure 3:**
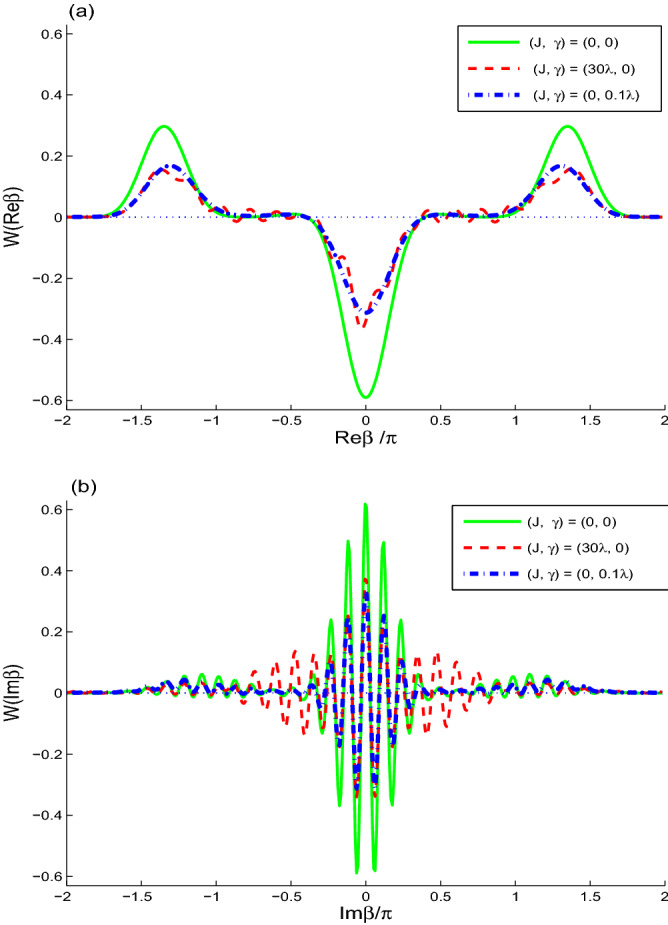
Different color curves show the effects of the two-qubit coupling and the ID rates on the partial Wigner functions $W(\text {Re}\beta )$ and $W(\text {Im}\beta )$. They are plotted for different cases $(J, \gamma )=(0, 0)$ (solid curves), $(J, \gamma )=(30\lambda , 0)$ (dash curves), $(J, \gamma )=(0, 0.1\lambda )$ (dash-dot curves) with $\alpha =4$ at $\lambda t=\pi$.

**Figure 4:**
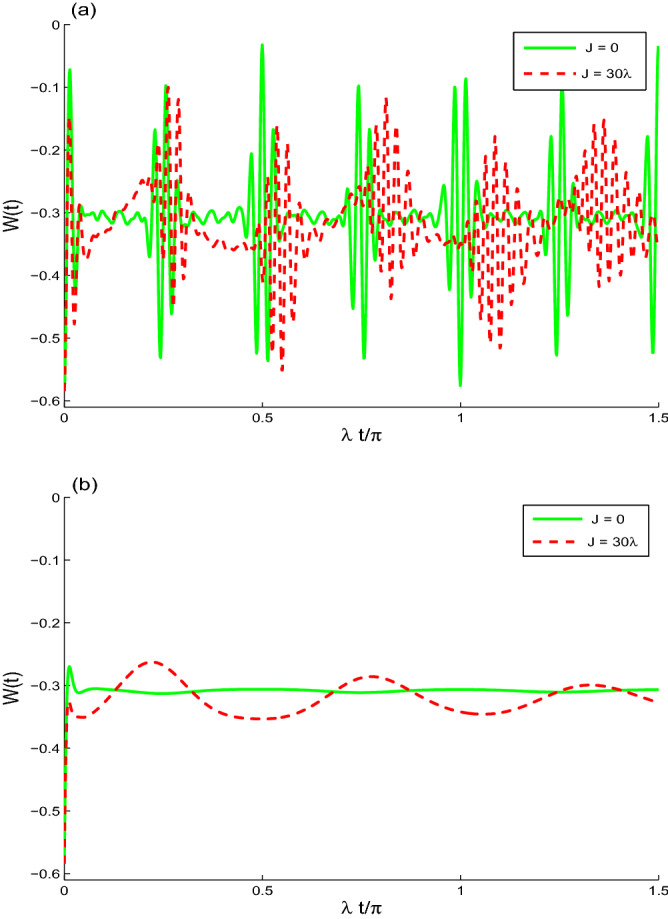
Wigner function *W*(*t*) for $\alpha =4$ when $\gamma =0.0$ in (**a**) and $\gamma =0.01\lambda$ in (**b**) with different cases $J=0$ (sold curves), $J=30\lambda$ (dash curves).

In Figs. 1, 2, 3 and 4, the Wigner function $$W(\beta )$$$$(\beta =\text {Re}\beta +i\text {Im}\beta )$$ and its partial functions [as: $$W(\text {Re}\beta )$$, $$W(\text {Im}\beta )$$ and *W*(*t*)] of the cavity-filed system are plotted to probe the effects of the different physical parameters; Namely the effect of the non-linear interaction between the two-qubit and the cavity field, the interaction between the qubits and the intrinsic decoherence.

In Fig. 1, the behavior of the Wigner function $W(\beta )$ of the initial even coherent state, $\frac{1}{A}[|\alpha \rangle +|-\alpha \rangle ]$, is displayed in the phase space. In Fig. 1a, WF has symmetrical maximum and minimum. The negative and positive parts of the Wigner distribution are clearly detectable in the phase space. The negativity is the natural signature of the non-classicality.

The non-classicality disappears for larger time (see Fig. 1b).

We deduce from Fig. 2a that the generated negativity and positivity are enhanced by increasing the coupling between the two qubits. From Fig. 2b, we observe that the intrinsic decoherence has a clear effect on the Wigner distribution. The negativity and positivity of the $W(\beta )$ function are reduced.

In Fig. 3, we plot the partial functions $W(\text {Re}\beta )$ at fixed value $\text {Im}\beta =0.06127\pi$ (see Fig. 3a), and $W(\text {Im}\beta )$ at fixed value $\text {Re}\beta =0$ (see Fig. 3b). This to analyze the behavior of the minimum of $W(\beta )$ (at $\beta _{\text {MV}}=0 + 0.06127\pi i$).

Solid curves of Fig. 3a show the behavior of the Wigner function $W(\text {Re}\beta )$ against the real component of complex space $\beta$.

The Wigner function presents a pronounced non-classicalty propriety around $\text {Re}\beta =0$, while it is purely classical out of that interval. By increasing the coupling *J* or the intrinsic decoherence the distinction between the classical and the quantum behaviors of the Wigner function become more noticeable.

Figure 3b, shows the dependence of the Wigner function on the imaginer component of $\beta$ in the solid curve of $W(\text {Im}\beta )$. It shows damped oscillator behavior around the origin. This indicates a high sensibility of the nonclassical/classical behaviors of the Wigner function to the imaginary part of the phase space parameter $\beta$.

Figure 4 displays the dynamics of the minimum of the Wigner function: $W(t)=W(\beta _{\text {LN}},t)$ ($\beta _{\text {LN}}=0.009296\pi -0.06127\pi i$ (see Fig. 1a)). The dynamics of the Wigner function *W*(*t*) is quasi-periodic. We observe that: (1) The Wigner function is non classical. (2) The coupling between the two qubits leads to the increase of the negativity of *W*(*t*) with pronounced oscillations. (3) The intrinsic decoherence rate stabilizes the dynamics of the Wigner function to its stationary state (see Fig. 4b). The negativity is hypersensitive to the intrinsic decoherence and the coupling between the two qubits.

## Q-distribution

### Phase space information of Wehrl density (WD)

The Wehrl density is one of the applications of the Q-distribution that is used to investigate the phase space information in the coupled two qubit system. The phase space information is determined by the angles $\theta$ and $\phi$^30,31^. When the information is lost, the Wehrl density is independent of the phase space angles.

**Figure 5:**
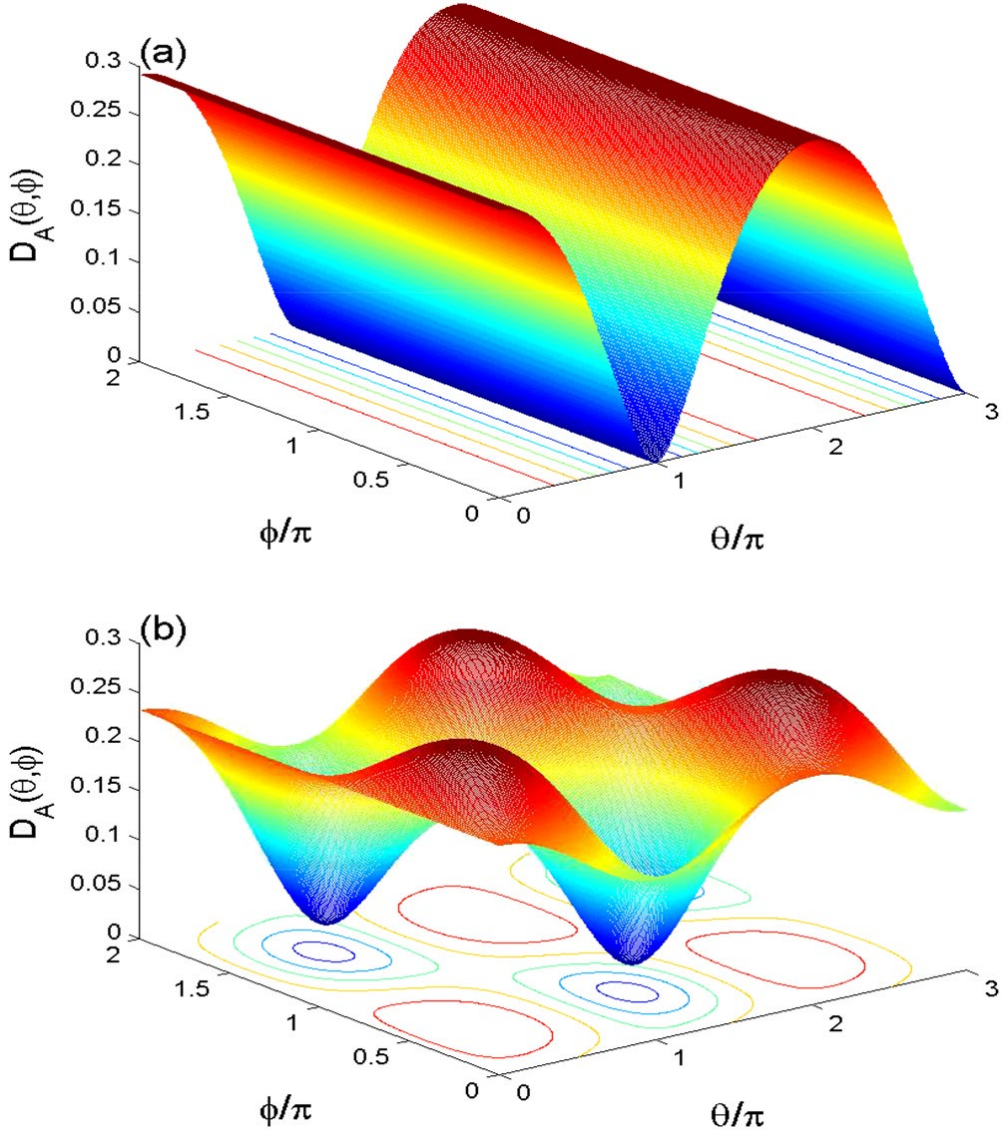
The behavior of the partial Wehrl density $D_{A}(\theta ,\phi )$ in the phase space. It is plotted at $\lambda t=0$ in (**a**) $\lambda t=2.011\pi$ in (**b**) for $\alpha =4$ with the cases $(\Omega , \gamma )=(0, 0)$.

Using the basis: $\{|\varpi _{i}\rangle \}$, the two-qubit Bloch coherent states can be written as^36^:

13 $$\begin{aligned} \!|\Phi \rangle _{12}\!&=\cos \frac{\theta _{1}}{2}\cos \frac{\theta _{2}}{2}|\varpi _{1}\!\rangle +e^{i\phi _{2}}\cos \frac{\theta _{1}}{2}\sin \frac{\theta _{2}}{2} |\varpi _{2}\rangle \\&\quad +\!e^{i\phi _{1}}\sin \frac{\theta _{1}}{2} \cos \frac{\theta _{2}}{2}|\varpi _{3}\!\rangle +e^{i(\phi _{1}+\phi _{2})}\sin \frac{\theta _{1}}{2} \sin \frac{\theta _{2}}{2}|\varpi _{4}\!\rangle .\nonumber \end{aligned}$$

Consequently, the QF of the two-qubit system $\rho ^{Q_{s}}(t)$ is

14 $$\begin{aligned} Q_{12}(\Phi ,t)= & {} \frac{1}{4\pi ^{2}}\langle \Phi |\rho ^{Q_{s}}(t)|\Phi \rangle _{12}, \end{aligned}$$

and its partial QFs of the *k*-qubit (for example for $k=1$) is given by

15 $$\begin{aligned} Q_{1}(\theta _{1},\phi _{1},t)= & {} \int ^{\pi }_{0}\int ^{2\pi }_{0} Q_{12}(\Phi ,t) \sin \theta _{2}\,d\phi _{2}\, d\theta _{2}. \end{aligned}$$

The partial Wehrl density of the *k*-qubit, $D_{k}(\theta ,\phi ,t)$, is given by

16 $$\begin{aligned} D_{k}(\theta ,\phi ,t)=-Q_{k}(\theta ,\phi ,t)\,\ln [Q_{k}(\theta ,\phi ,t)]. \end{aligned}$$

**Figure 6:**
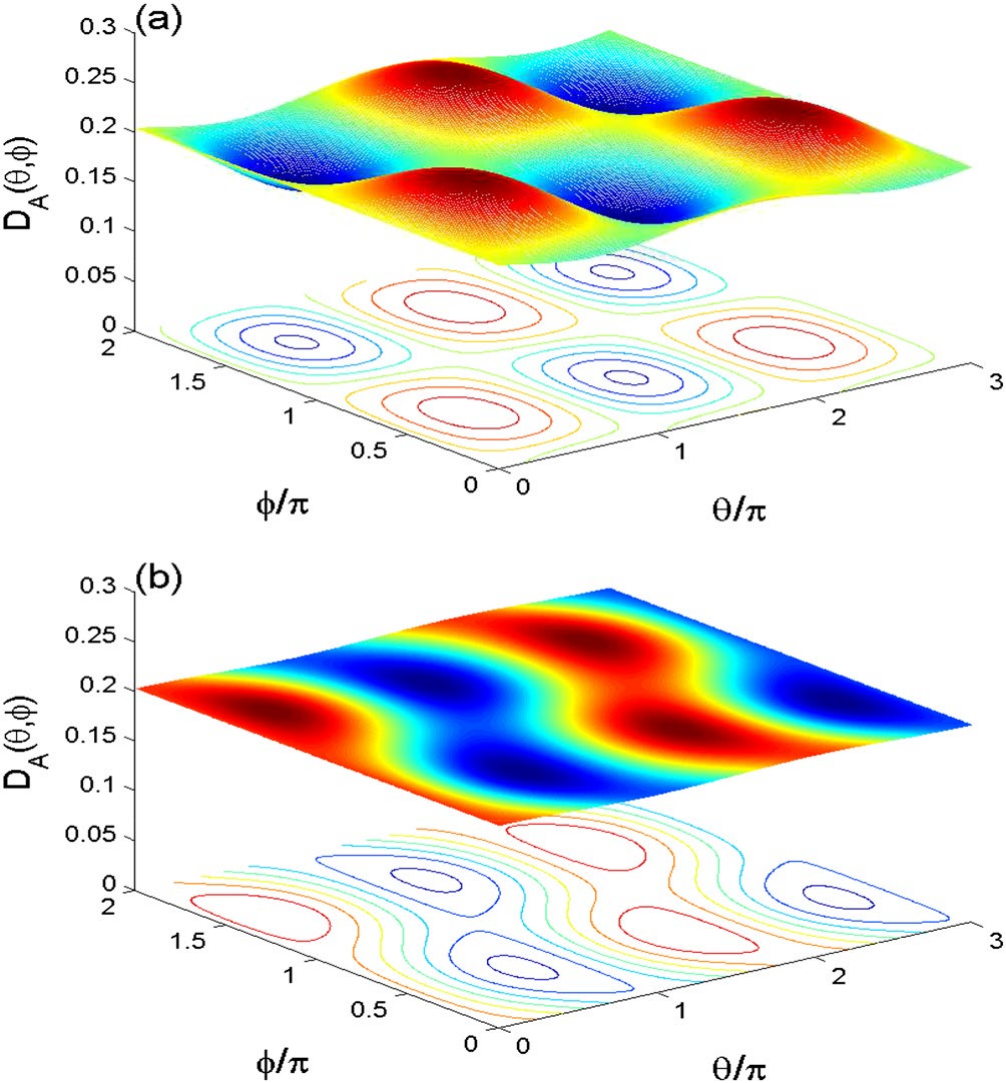
Wehrl density $D_{A}(\theta ,\phi )$ in the phase space at $\lambda t=2.011\pi$ with $\alpha =4$ for $(J, \gamma )=(30\lambda , 0)$ in (**a**), and $(J, \gamma )=(0, 0.01\lambda )$ in (**b**).

**Figure 7:**
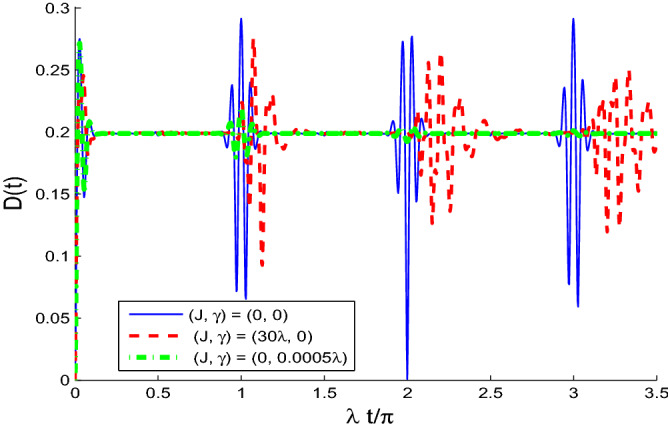
The dynamical behavior of the partial Wehrl density *D*(*t*) for $(\theta ,\phi )=(\pi ,\pi )$ and $\alpha =4$ for $(J,\gamma )=(0,0)$ (solid curve) $(J,\gamma )=(30\lambda , 0)$(dashed curve) and $(J,\gamma )=(0, 0.0005\lambda )$(dashed-doted curve).

Figures 5, 6 and 7 show the effects of the non-linear interaction between the two-qubit and the cavity field, the interaction between the qubits and the intrinsic decoherence, on the partial Wehrl density of the *A*-qubit $D_{A}(\theta ,\phi )$.

In Fig. 5, the partial Wehrl density $D_{A}(\theta ,\phi )$ is plotted at $\lambda t=0$ and $\lambda t=2.011\pi$ in the phase space for $\alpha =4$ and $(J,\gamma )=(0, 0)$. We note that the WD function has regular oscillatory surface with $2\pi$-period (see Fig. 5a). Figure 5b, at $\lambda t=2.011\pi$, illustrates that the pecks and bottoms of the partial Wehrl density $D_{A}(\theta ,\phi )$ are regularly distributed. The contour plots of the WD function confirm the dependence of the heights and depths of the phase space WD distribution on the angular variables $\theta$ and $\phi$.

Figure 6a, shows that the phase space information of the Wehrl density can be controlled by the two-qubit interaction coupling. We note from Fig. 6b that the generated pecks and bottoms of the WD disappear completely due to the intrinsic decoherence.

Based on the fact that the partial Wehrl density $D_{A}(\theta ,\phi )$ has zero-value at $\theta =\pi$, we displays the dynamics of the Wehrl density $D(t)=D_{A}(\theta ,\phi )$ in Fig. 7 for $(\theta , \phi )=(\pi , \pi )$. We observe that the dynamics of the Wehrl density has regular oscillations with $2\pi$-period. The coupling rate of the two-qubit interaction leads to smoothing and reduction of the WD oscillations. It disappears completely in the presence of the intrinsic decoherence.

### Wehrl entropy

In the phase space $\theta \in [0, \pi ]$ and $\phi \in [0, 2\pi ]$, we can investigate the purity loss by using the Wehrl entropy^28^. It is a good measure to the entanglement in the sparable state and the phase space purity-loss in the mixed sate, which are useful tools in quantum information^51^. The partial Wehrl entropies of the *k*-qubit are given by

17 $$\begin{aligned} \!\!\!\!S_{k}(t)= & {} \int _{0}^{2\pi }\int _{0}^{\pi } D_{k}(\theta ,\phi ,t)\,\sin \theta \;d\theta \,d\phi . \end{aligned}$$

Without loss of generality, we study the Wehrl entropy coherence loss of the *A*-qubit by the function $S_{A}(t)$. If the two qubits are initially prepared in the state $|e_{A}e_{B}\rangle$,

18 $$\begin{aligned} S_{A}(0)&=-2\int _{0}^{\pi /2} \sin 2\theta \cos ^{2}\theta \ln [\cos ^{2}(\theta )/2\pi ]\;d\theta \nonumber \\&=2.3379. \end{aligned}$$

Therefore, the boundary values of the function $S_{A}(t)$ is^30,36^,

19 $$\begin{aligned} 2.3379 \le S_{A}(t)\le \ln (4\pi ). \end{aligned}$$

For the case of one-qubit system, there is a relation between the Wehrl entropy and the other entropies as: von Neumann entropy and linear entropy^52^. It was proved that they exhibit similar behaviors. The Wehrl entropy can be determined from phase-space distribution. While the von Neumann entropy is calculated from the reduced density matrix.

**Figure 8:**
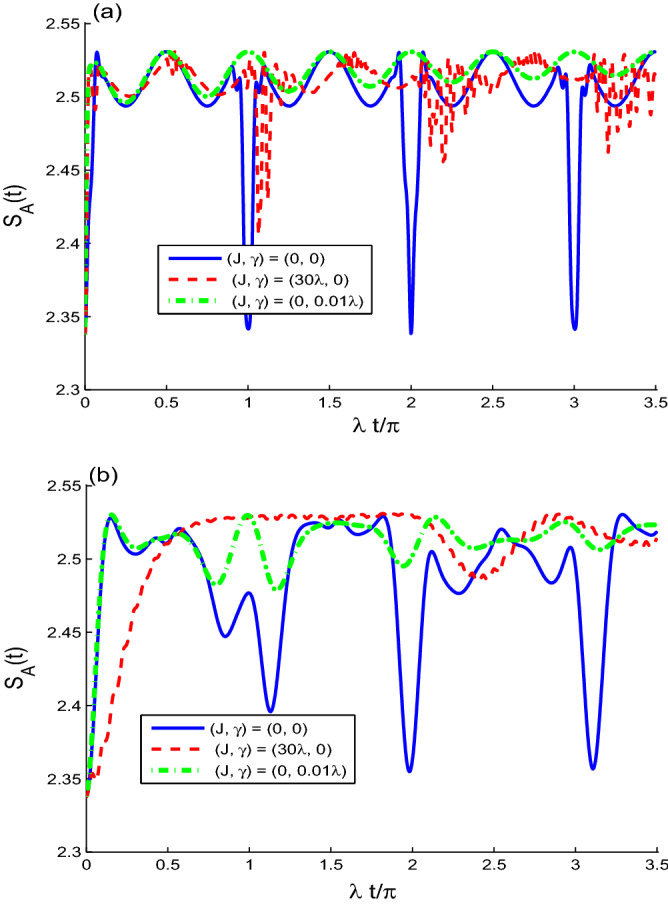
The dynamical behavior of the Wehrl entropy $S_{A}(t)$ for different cases $\alpha$: $\alpha =4$ in (**a**) and $\alpha =1$ in (**b**). For $(J, \gamma ) = (0, 0)$ (solid curves), $(J, \gamma ) = (30\lambda , 0)$ (dashed curves) and $(J, \gamma ) = (0, 0.01\lambda )$ (dashed-doted curves).

The Wehrl entropy, $S_{A}(t)$ is used to measure the mixedness of the *A*-qubit. In Fig. 8a, the function $S_{A}(t)$ is plotted for large value $\alpha =4$ and for different sets of parameters: $(J, \gamma )=(0, 0)$, $(J, \gamma )=(30\lambda , 0)$ and $(J, \gamma )=(0, 0.01\lambda )$. In the absence of the intrinsic decoherence, the Wehrl entropy $S_{A}(t)$ oscillates quasi-periodically with a $\pi$-period. The *A*-qubit is in mixed state.

Dashed curves of Fig. 8 shows how the coupling between the two qubits, $J/\lambda$, affects the mixedness of the qubit state. The coupling constant *J* improves the mixedness of the qubit A. In the presence of the intrinsic decoherence, the mixdness of the A-qubit is enhanced. We also observe that the amplitudes, the regularity and the stability of the generated mixedness can be affected by the initial coherent field intensity.

## Conclusion

In this investigation, we have explored analytically, two identical qubits. The two qubits are in resonant and in nonlinear interaction with a quantum field. The positivity and negativity of the Wigner distribution are explored to analyze the non-classicality. The intrinsic decoherence and the coupling between the two qubits lead to notable changes in the dynamical behavior of the non-classicality. The phase space information and the quantum coherence relay on the physical parameters. The generated mixedness can be improved by increasing the coupling between the two qubits. The growth of the Wehrl entropy, due to the cavity-qubits interaction, is enhanced by the increase of the intrinsic decoherence. The control of the non-classicality and the quantum coherence opens the door to the conception of optical states with unconventional proprieties.

Recently, the non-classicality and the quantum coherence were used to realize quantum computations^53,54^, quantum tomography^55^, quantum interference^56^ as well as to implement large cat states in finite-temperature reservoir^57^.
