# Author Correction: Quasi-probability information in a coupled two-qubit system interacting non-linearly with a coherent cavity under intrinsic decoherence

**DOI:** 10.1038/s41598-020-75422-w

**Published:** 2020-11-20

**Authors:** Abdel‑Baset A. Mohamed, Hichem Eleuch

**Affiliations:** 1 Department of Mathematics, College of Science and Humanities in Al-Afaj, Prince Sattam bin Abdulaziz University, Al-Afaj, Saudi Arabia; 2 Department of Mathematics, Faculty of Science, Assiut University, Assiut, Egypt; 3 Department of Applied Sciences and Mathematics, College of Arts and Sciences, Abu Dhabi University, Abu Dhabi, UAE; 4 Institute for Quantum Science and Engineering, Texas A&M University, College Station, TX 77843 USA

Correction to: *Scientific Reports* https://www.doi.org/10.1038/s41598-020-70209-5, published online 06 August 2020

The original version of this Article contained a typographical error in the title of the paper,

“Quasi-probability information in an coupled two-qubit system interacting non-linearly with a coherent cavity under intrinsic decoherence”

Now reads:

“Quasi-probability information in a coupled two-qubit system interacting non-linearly with a coherent cavity under intrinsic decoherence”

This error has now been corrected in the PDF and HTML versions of the Article.

